# Evaluation of Circulating MicroRNAs and Adipokines in Breast Cancer Survivors with Arm Lymphedema

**DOI:** 10.3390/ijms231911359

**Published:** 2022-09-26

**Authors:** Khairunnisa’ Md Yusof, Kira Groen, Rozita Rosli, Maha Abdullah, Rozi Mahmud, Kelly A. Avery-Kiejda

**Affiliations:** 1 School of Biomedical Sciences and Pharmacy, College of Medicine, Health and Wellbeing, The University of Newcastle, Newcastle, NSW 2308, Australia; 2 Hunter Medical Research Institute, New Lambton Heights, NSW 2305, Australia; 3 Department of Biomedical Sciences, Faculty of Medicine and Health Sciences, Universiti Putra Malaysia, Serdang 43400, Selangor, Malaysia; 4 Department of Pathology, Faculty of Medicine and Health Sciences, Universiti Putra Malaysia, Serdang 43400, Selangor, Malaysia; 5 Department of Radiology, Faculty of Medicine and Health Sciences, Universiti Putra Malaysia, Serdang 43400, Selangor, Malaysia

**Keywords:** microRNA, breast cancer survivor, arm lymphedema, BCRL, metabolic syndrome, molecular markers, adipokines

## Abstract

Breast cancer-related lymphedema (BCRL) is a form of secondary lymphedema that is characterized by abnormal swelling of one or both arms due to the accumulation of lymph fluid in the interstitial tissue spaces, resulting from obstruction of the lymphatic vessels due to surgery insults, radiotherapy, or chemotherapy. Due to the multifactorial nature of this condition, the pathogenesis of secondary lymphedema remains unclear and the search for molecular factors associated with the condition is ongoing. This study aimed to identify serum microRNAs and adipokines associated with BCRL. Blood was collected from 113 breast cancer survivors and processed to obtain serum for small RNA-sequencing (BCRL vs. non-BCRL, *n* = 7 per group). MicroRNAs that were differentially expressed (fold change >1.5, *p* < 0.05) between lymphedema cases and those without lymphedema were further quantified in a validation cohort through quantitative reverse transcription PCR (BCRL *n* = 16, non-BCRL, *n* = 83). Leptin and adiponectin levels were measured in a combined cohort (BCRL *n* = 23, non-BCRL *n* = 90) using enzyme-linked immunosorbent assays. Two of the most significantly upregulated microRNAs, miR-199a-3p and miR-151a-3p, were strongly correlated with the onset of lymphedema and diabetes mellitus in the BCRL group. Leptin levels were higher in the BCRL cohort compared to the non-BCRL cohort (*p* < 0.05). A metabolic syndrome biomarker, the adiponectin/leptin ratio, was found to be lower in the BCRL group than in the non-BCRL group (median: 0.28 vs. 0.41, *p* < 0.05). Extensive studies on the mechanisms of the identified microRNAs and association of leptin with arm lymphedema may provide new insights on the potential biomarkers for lymphedema that should be followed up in a prospective cohort study.

## 1. Introduction

Breast cancer mortality rates have steadily declined over the years due to early detection, improved treatment, and better management. According to GLOBOCAN (2018), the 5-year net survival rates of breast cancer in high income countries (Australia, the United States, Japan, and South Korea) and in the European region are more than 85% [1]. Meanwhile, the survival rates of breast cancer in Malaysia are 89.7% for 1-year and 66.8% for 5-year [2]. Although the survival rates for breast cancer are increasing, cancer survivors face several challenges following cancer treatment including arm lymphedema [3,4]. Arm or secondary lymphedema is characterized by abnormal swelling of one or both arms due to an accumulation of lymph fluid in the interstitial tissue spaces following surgery-associated damage of the lymphatic vessels such as axillary lymph nodes dissection, mastectomy, and regional radiotherapy [5,6]. Due to its multifactorial nature, the pathogenesis of secondary lymphedema remains unclear and the search for molecular factors associated with this condition is still ongoing.

MicroRNAs (miRNAs) are short non-coding RNAs of ~18 to 20 nucleotides that are stable and detectable in bodily fluids such as serum, plasma, and urine [7]. MicroRNAs mediate gene expression regulation and, thus, are involved in biological processes including cell proliferation, apoptosis, adipogenesis, and angiogenesis. Due to their functionally active properties and stability within vesicles, miRNAs have been extensively studied as potential biomarkers in various diseases such as Alzheimer’s [8,9], cancers [10], and cardiovascular diseases [11,12]. To our knowledge, an understanding of the role of miRNAs in secondary lymphedema remains to be explored, but evidence from related pathological conditions indicates their potential involvement [13]. For instance, miR-29 has been found to be a negative regulator of skin fibrosis, one of the hallmarks of lymphedema. It was reported that miR-29 inhibited the synthesis of elastin and fibrillin by negatively regulating transforming growth factor-beta (TGF-β) and, thus, increasing the action of matrix metalloproteinase-1, an enzyme that breaks down the extracellular matrix and thereby prevents the formation of fibrous tissue [14].

Mounting evidence has identified that a high body mass index (BMI) is one of the novel risk factors for secondary lymphedema and we have corroborated this factor in our previous work [15]. We have also demonstrated that hypertension and diabetes mellitus are confounding factors associated with secondary lymphedema in breast cancer survivors. A recent study suggested that secondary lymphedema has been linked to metabolic syndrome (MetS) due to a significant association between insulin resistance and central obesity in 28 breast cancer patients with lymphedema [16]. In support of this, higher serum leptin levels and dysregulation of the adipocytokine signalling pathway are associated with lymphedema [17], further strengthening the association of altered lipid transport and fat deposition within lymphedema. This notion, however, needs to be validated at the molecular level and in a larger cohort.

Therefore, the characterization of BCRL-associated miRNAs and adipokines may facilitate the discovery of novel molecular factors involved in the pathophysiology of this condition. In the present study, the serum expression of miRNAs and the level of adipokines was explored and correlated with patients’ characteristics to provide new insights on the mechanisms that orchestrate secondary lymphedema in breast cancer survivors.

## 2. Results

### 2.1. Characteristics of Study Participants

The study was comprised of two cohorts that were used for microRNA analysis (a) discovery cohort, consisting of healthy control (HC, *n* = 7), breast cancer without lymphedema (non–BCRL, *n* = 7), and breast cancer with lymphedema patients (BCRL, *n* = 7). The second cohort, (b) the validation cohort, was comprised of non–BCRL (*n* = 83) and BCRL patients (*n* = 16). Meanwhile, a combination of both cohorts (combined cohort), consisting of BCRL (*n* = 23) and non-BCRL (*n* = 90), was used for adipokine analysis. The characteristics of participants in the discovery and validation cohort were detailed in the Supplementary Material (Supplementary Table S1). Analysis of the combined cohort (Table 1) showed that the average of all breast cancer participants in the study was 51.78 (±8.33) years, with a mean period following breast cancer diagnosis of 5.77 (±4.39) years and a mean BMI of 28.29 (±5.93) kg/m^2^. There were no significant differences between BCRL and non-BCRL in age, years after diagnosis, waist-to-hip ratio (WHR), fat percentage, blood pressure, pulse rate, and the Functional Assessment of Cancer Therapy–Breast (FACT-B) scores. Following that, the significant difference of BMI and WHR between the two groups also subsided in the combined cohort (*p* > 0.05). There was a statistically significant difference between BCRL and the non-BCRL group for waist circumference (median: 98.5 vs. 89.6, *p* = 0.049), waist-to-height ratio (WHtR, median: 0.62 vs. 0.57, *p* = 0.049), and the Disabilities of the Arm, Shoulder, and Hand (DASH) scores (median: 40.00 vs. 17.37, *p* < 0.001) (Table 1). There were more women with hypertension (43.5% vs. 16.7%, OR = 3.85, 95% C.I = 1.14–10.35, *p* = 0.006) and diabetes mellitus (30.4% vs. 11.1%, OR = 3.50, 95% C.I = 1.16–10.57, *p* = 0.021) in the BCRL group than in the non-BCRL group.

### 2.2. Circulating miRNAs Are Associated with Lymphedema Development in Breast Cancer Survivors

**Figure 1:**
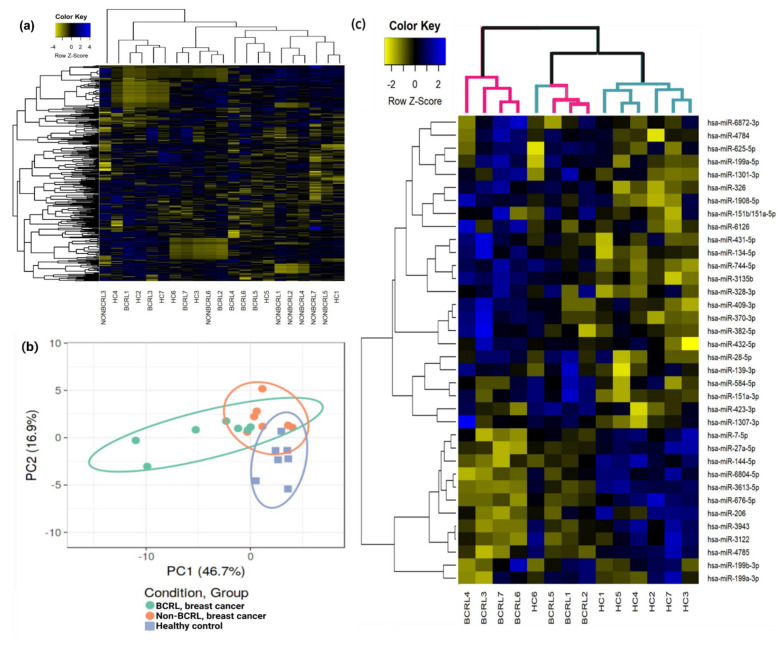
Analysis of serum miRNAs of HCs, non-BCRL, and BCRL by miRNA-sequencing. (**a**) Hierarchical clustering of the 960 detected miRNAs across all groups. Similarity in the expression patterns between miRNAs (branches shown on the left-hand panel) was measured using Pearson’s correlation. miRNAs are colored according to their expression level, where upregulated expression is represented by blue, downregulated expression is represented by yellow, and equal expression is represented by black. Distances between clustered branches represent the average distances between miRNAs and samples in the cluster (bottom panel). The height of each branch represents the degree of similarity within the cluster. (**b**) A PCA plot demonstrating the average miRNA expression patterns of BCRL (green dots, green circle), non-BCRL (orange dots, orange circle), and HCs (blue-squares, blue circle). The X-axis indicates PCA Component 1 (PC1: 46.7% variance) and the Y-axis shows PCA Component 2 (PC2: 16.9% variance). (**c**) Differentially expressed miRNAs in BCRL vs. HCs. Supervised hierarchical cluster analysis was performed on 36 miRNAs significantly altered between BCRL (pink branches, top) and HCs (green branches, top). Similarity in the expression patterns between miRNAs (branches shown on the left-hand panel) was measured using Pearson’s correlation. Distances between clustered branches represent the average distances between miRNAs (right-hand panel) or individual samples (bottom panel) within the cluster. The height of each branch represents the degree of similarity within the cluster. The bottom panel indicates individual samples from BCRL and HC groups. miRNAs are colored according to their expression level, where upregulated expression is represented by blue, downregulated expression is represented by yellow, and equal expression is represented by black. HC healthy controls; BCRL breast cancer with lymphedema.

In order to determine miRNAs that may potentially serve as biomarkers for BCRL, miRNA profiling was performed by small RNA-sequencing using total RNA extracted from the serum. Serum miRNAs were compared between the non-BCRL and BCRL groups, as well as to HCs (Figure 1). In this analysis, 960 miRNAs were detected in at least one of these groups. As shown in the hierarchical clustering (Figure 1a), there was no change in global miRNA expression between the groups. The principal component analysis (PCA) plot further confirmed that there were no differences in the overall expression of miRNAs between the BCRL and non-BCRL group. However, the PCA was able to separate breast cancer cases from the HC group (Figure 1b).

Following this analysis, individual, differentially expressed miRNAs between BCRL cases and HCs, as well as non-BCRL cases and HCs, were identified. As shown in Figure 1c, a total of 36 miRNAs were differently expressed between BCRL cases and HCs and hierarchical clustering of these miRNAs was able to separate the two groups. Six HCs were clustered together, while one HC sample clustered with the BCRL group, indicating overlapping miRNA expression patterns. Of the 36 miRNAs, 10 miRNAs were downregulated (FC < 0.5) and 26 miRNAs were upregulated (FC > 2.0) in BCRL. Table 2 details the differentially expressed miRNAs between BCRL versus HCs.

**Figure 2:**
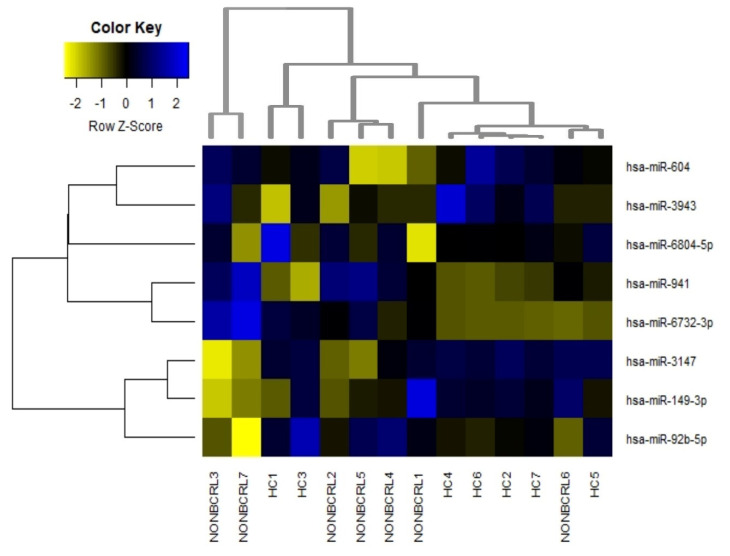
Differentially expressed miRNAs in non-BCRL vs. HCs. Supervised hierarchical cluster heatmaps were performed on eight miRNAs significantly altered between non-BCRL and HCs. Similarity in the expression patterns between miRNAs (branches shown on the left-hand panel) was measured using Pearson’s correlation. Distances between clustered branches represent the average distances between miRNAs (right-hand panel) and individual samples in the cluster (bottom panel). The height of each branch represents the degree of similarity within the cluster. MiRNAs are colored according to their expression level, where upregulated expression is represented by blue, downregulated expression is represented by yellow, and equal expression is represented by black. HC healthy controls; non-BCRL breast cancer without lymphedema.

Further, miRNA analysis revealed eight miRNAs to be differentially expressed between non-BCRL and HCs (Figure 2 and Table 3). The number of differentially expressed miRNAs were much lower compared to BCRL vs. HC samples. Moreover, there was no clear separation of the differentially expressed miRNAs between non-BCRL and HCs (Figure 2), suggesting the miRNA expression profiles were similar between the two groups. Out of eight miRNAs, seven were downregulated (FC < 0.5) and one miRNA was upregulated (FC > 2.0).

**Figure 3:**
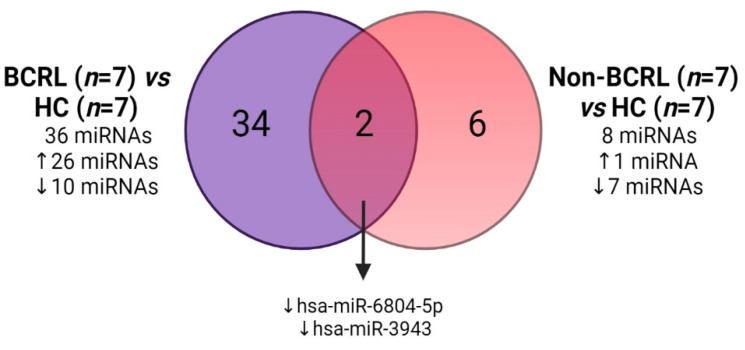
Venn diagram representing overlapping miRNA expression between BCRL vs. HCs and non-BCRL vs. HCs. A total of 36 miRNAs were differentially expressed in the BCRL group (purple and left-hand circle) and 8 miRNAs were differentially expressed in the non-BCRL vs. HCs (8 miRNAs, red and right-hand circle). The direction of the regulation is represented with an upward pointing arrow (upregulated) and a downward pointing arrow (downregulated).

To define miRNAs that were specifically modified in breast cancer survivors who had lymphedema, Venn diagram analysis was performed on the miRNAs that were differentially expressed in the BCRL or non-BCRL group when compared to the HCs. It was reasoned that miRNAs that were differentially expressed in the BCRL cases and not in the non-BCRL cases may serve as biomarkers for BCRL. In this analysis (Figure 3), 34 miRNAs were uniquely expressed in BCRL vs. HCs when compared to only six miRNAs that were uniquely expressed in non-BCRL vs. HCs. The expression of two miRNAs overlapped in BCRL and non-BCRL vs. HCs, including miR-6804-5p and miR-3943, and the direction of their regulation was concordant.

### 2.3. Verification and Validation of the Differential Expression of miR-199a-3p and miR-151a-3p in BCRL by qPCR

**Figure 4:**
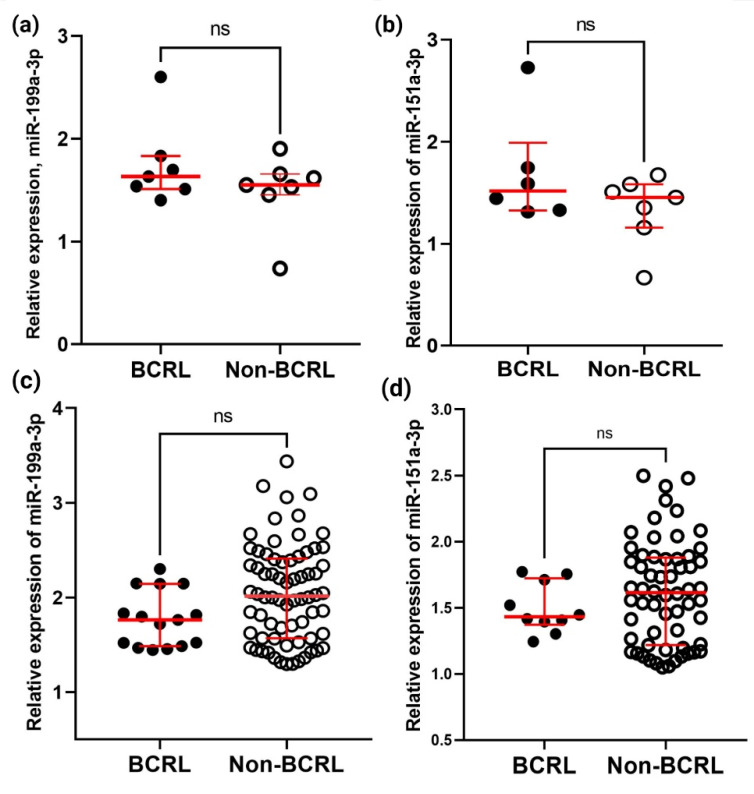
Relative expression of miR-199a-3p and miR-151a-3p in the discovery and combined cohort as measured by qPCR. Relative expression of the miRNAs was calculated using the 2^−∆∆Ct^ method and were compared between the BCRL and non-BCRL groups. (**a**) The expression of miR-199a-3p and (**b**) miR-151a-3p were validated in the discovery cohort (BCRL, *n* = 7 and non-BCRL, *n* = 7); (**c**) the relative expression of miR-199a-3p and (**d**) miR-151a-3p in samples of the validation cohort. Open dots represent BCRL cases (*n* = 16) and black dots represent cases in the non-BCRL group (*n* = 90). Data were normalized to an endogenous control, miR-16-5p. All samples were analyzed in triplicate. *p*-values were calculated using Mann–Whitney test (*p* < 0.05), with ns denoting a non-significant difference. Results are expressed as the median and error bars denote interquartile range or IQR.

Two of the miRNAs with the highest average read counts, miR-199a-3p and miR-151a-3p, were selected for subsequent analysis. In addition to having the highest read counts, these miRNAs were significantly different in comparisons of BCRL with healthy controls (Table 2) in the discovery cohort RNA sequencing analysis, but not different in comparisons of non-BCRL with healthy controls (Table 3), highly suggestive of their involvement in BCRL development. The expression of miR-199a-3p and miR-151a-3p was quantified in the discovery cohort by qPCR to verify the small RNA-sequencing findings. The expression level of miR-199a-3p was quantified in all cases but the expression of miR-151a-3p could only quantified in six samples as qPCR failed due to low Ct values (undetermined) in one of the samples. As shown in Figure 4, there was no significant difference in the level of expression of miR-199a-3p or miR-151a-3p between BCRL and non-BCRL cases (Figure 4a,b).

The expression of miR-199a-3p and miR-151a-3p was also quantified in a validation cohort comprising 99 serum samples with BCRL (*n* = 16) and non-BCRL (*n* = 83). miR-199a-3p expression was detected in 15/16 BCRL and 70/83 non-BCRL cases, whereas the expression of miR-151a-3p was detected in 10/16 BCRL and 60/83 non-BCRL cases. The expression of miR-199a-3p was higher in non-BCRL (median: 2.01, IQR: 0.84) compared to BCRL (median: 1.76, IQR: 0.67, *p* = 0.054). Similarly, the trend of expression in miR-151a-3p was higher in non-BCRL (median: 1.62, IQR: 0.67) compared to BCRL (median: 1.43, IQR: 0.35, *p* = 0.374). However, neither of these results were statistically significant (Figure 4c,d). One of the reasons for the lack of agreement between the miRNA-sequencing and the qPCR data is the differences in the way the data were analyzed. Venn diagram analysis of the miRNA-sequencing data identified miRNAs which were differentially expressed in the BCRL vs. HC and non-BCRL vs. HC. However, this does not necessarily mean that they were significantly different between BCRL and non-BCRL cases. Meanwhile, for the qPCR data, the relative expression of miR-199a-3p and miR-151a-3p was compared between BCRL and non-BCRL groups directly.

**Figure 5:**
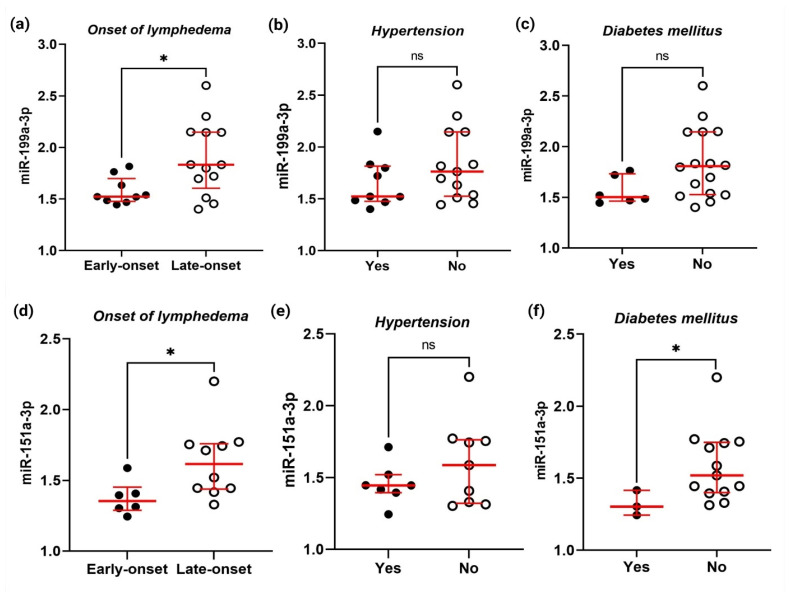
The relative expression of miR-199a-3p and miR-151a-3p in the BCRL group (*n* = 23) stratified by the onset of lymphedema, hypertension, and diabetes mellitus status. Relative expression of miR-199a-3 by qPCR analyzed according to (**a**) onset of lymphedema, (**b**) hypertension, and (**c**) diabetes mellitus status. Relative expression of miR-151a-3p stratified by (**d**) onset of lymphedema, (**e**) hypertension, and (**f**) diabetes mellitus. Samples were measured in triplicate and results are shown as a dot plot of the relative normalized expression of the miRNA of interest (2^−∆∆Ct^). * *p*-values were calculated by Mann–Whitney test (*p* < 0.05), with ns denoting a non-significant difference. The horizontal bars represent pairwise comparison between respective variables and dichotomous outcomes, blue lines represent the median and error bars indicate the IQR of each group.

Given that hypertension was determined to be a BCRL confounding factor in the study population and diabetes mellitus was associated with the onset of lymphedema [15], the expression of the miRNAs (miR-199a-3p and miR-151a-3p) was analyzed according to the onset of lymphedema (early-onset lymphedema: persistent swelling within 12 months after breast surgery and late-onset lymphedema: swelling 12 months after breast cancer surgery), hypertension, and diabetes mellitus status. As shown in Figure 5, miR-199a-3p was significantly downregulated in early-onset lymphedema (median: 1.52, IQR: 0.22) when compared to the late-onset lymphedema group (median: 1.83, IQR: 0.54, *p* = 0.04).

Similarly, miR-151a-3p expression levels were lower in early-onset lymphedema (median: 1.35, IQR: 0.16) than in late-onset lymphedema (median: 1.61, IQR: 0.32, *p* = 0.010). No significant differences were found for either miRNA based on the hypertensive status. Additionally, BCRL subjects with diabetes mellitus demonstrate significantly lower miR-151a-3p expression (median: 1.50, IQR: 0) compared to those who were not diabetic (median: 1.52, IQR: 0.35, *p* = 0.039). Meanwhile, no significant differences were observed between diabetic cases with respect to miR-199a-3p expression levels (yes—median: 1.50, IQR: 0.27 vs. no—median: 1.81, IQR: 0.62, *p* = 0.058).

### 2.4. Functional Annotation Analysis of the Predicted Target Genes

**Figure 6:**
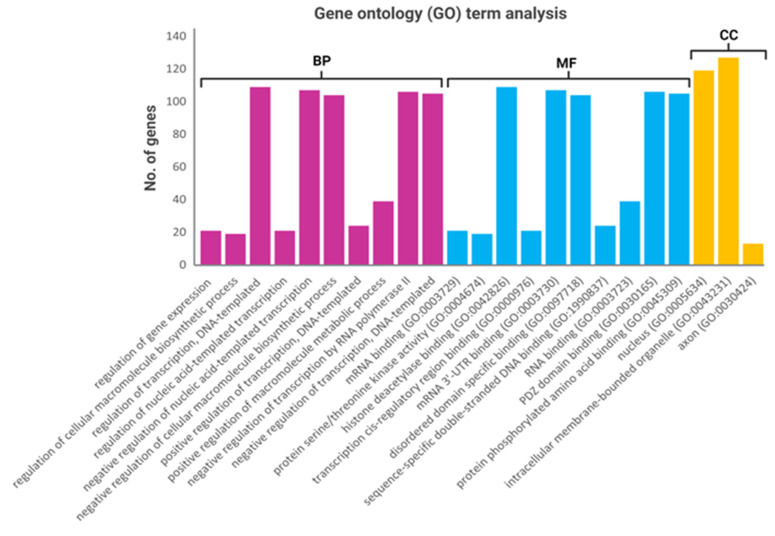
GO analysis of predicted target genes regulated by miR-151a-3p and miR-199a-3p. Data are presented as the number of genes (Y-axis) involved in each of the domains. The X-axis represents GO terms of each biological process (BP, purple bar), molecular function (MF, blue bar), and cellular component (CC, yellow bar) (X-axis) with FDR adjusted *p*-value < 0.05 (Benjamini–Hochberg).

Given that miR-199a-3p and miR-151a-3p were correlated with the onset of lymphedema and diabetes mellitus in BCRL cases, target prediction and gene set enrichment analyses were performed to define the potential functions of miR-199a-3p and miR-151a-3p in lymphedema. The prediction analysis revealed 320 target genes were correlated with either of the two miRNAs (Supplementary Table S2). Gene ontology (GO) analysis was predicted using the 320 miR-199a-3p and miR-151a-3p associated target genes. Results for the GO category analysis revealed the target genes were predominantly enriched in biological process terms including gene expression, regulation of transcription, and regulation of the metabolic process of macromolecules. Regarding the molecular function, the target genes were enriched in binding processes such as DNA, RNA, 3′-UTR and phosphorylated protein binding. Additionally, cellular component analysis revealed that the target genes were significantly enriched in the nucleus, intracellular membrane bound organelles, and axons (Figure 6 and Supplementary Table S3).

The analysis of the potential target genes regulated by miR-199a-3p and miR-151a-3p using the Kyoto Encyclopedia of Genes and Genomes (KEGG) online database revealed a broad spectrum of etiologies related to chronic diseases and inflammation. Of importance to the current study, this analysis showed that these target genes were significantly involved in pathways related to chronic lymphedema, including the TGF-β signalling pathway, phosphatidylinositol 3 kinase/protein kinase B (PI3K/Akt) signalling, and mitogen activated protein kinase (MAPK) signalling, which underscore the hallmark pathological features of lymphedema, namely fibrosis and inflammation. Additional pathways, such as extracellular matrix (ECM) receptor interaction, focal adhesion, regulation of actin cytoskeleton, and mammalian target of rapamycin (mTOR), are known to be involved in endothelial dysfunction. Additionally, sphingolipid and phospholipase D signalling, insulin resistance, dilated cardiomyopathy, cyclic guanosine monophosphate-protein kinase G (GMP-PKG) signalling, and hyperthrophic cardiomyopathy underscore the involvement of metabolic diseases that are predominant in subjects of the lymphedema group (Table 4 and Supplementary Table S4).

### 2.5. Circulating Leptin, Adiponectin, and Adiponectin/Leptin Ratio

Leptin and adiponectin are factors that are released by adipocytes, and they are highly associated with obesity [18,19]. Generally, circulating leptin concentrations are higher in obesity and metabolic syndromes, including type 2 diabetes, hypertension, or dyslipidemia, in conjunction with a decrease in adiponectin levels in the blood [20]. Consequently, the adiponectin/leptin ratio has been proposed as a predictive marker for adipose tissue dysfunction in obesity and cardiometabolic syndrome. Hence, these circulating adipokines were analyzed in the serum of breast cancer survivors to determine their potential as a biomarker for lymphedema; additionally, the adiponectin/leptin ratio was calculated to evaluate the degree of association of metabolic syndrome with BCRL.

**Figure 7:**
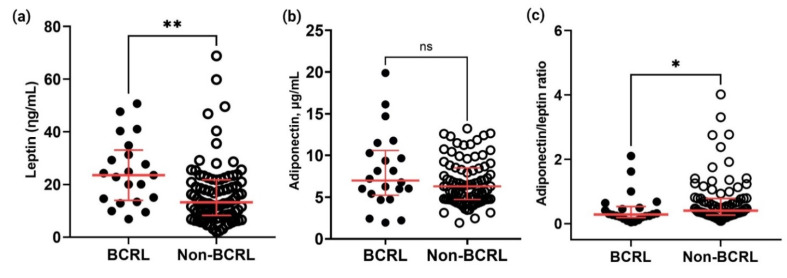
Comparison of the circulating leptin, adiponectin, and adiponectin/leptin ratio between the BCRL (*n* = 22) and non-BCRL group (*n* = 86). The levels of (**a**) leptin and (**b**) adiponectin were quantified by ELISA, whereas the (**c**) adiponectin/leptin ratio was calculated using a formula ((Adiponectin (μg/mL))/(Leptin (ηg/mL))). The horizontal black lines indicate a comparison line between the two groups and significant values were calculated by Mann–Whitney U test, *p* < 0.05. Asterisks in the figures represent statistical significance (* *p* < 0.05 and ** *p* < 0.001) and ns indicates non-significance. Samples were run in triplicate and the precision (CV%) of intra-assays were maintained between 10–15%. All data are presented as the median and error bars represent IQR.

As shown in Figure 7a, serum leptin concentrations were significantly higher in the BCRL group (n = 22, median: 23.9, IQR: 21.9) when compared to the non-BCRL group (n = 86, median: 13.2, IQR: 13.5, *p* = 0.010). However, the adiponectin concentration was not significantly different between the BCRL (median: 8.14, IQR: 5.36) and non-BCRL groups (median: 7.15, IQR: 3.86, *p* = 0.217) (Figure 7b). A significantly lower adiponectin/leptin ratio was identified in the BCRL group (median: 0.28, IQR: 0.34) compared to the non-BCRL group (median: 0.41, IQR: 0.52, *p* = 0.031). However, both groups exhibited a ratio of less than 0.5, suggesting a severe risk of developing metabolic syndrome in both BCRL and non-BCRL cases (Figure 7c).

### 2.6. Correlation Analysis of Circulating Adipokines with Relevant Characteristics in the BCRL Cohort

**Figure 8:**
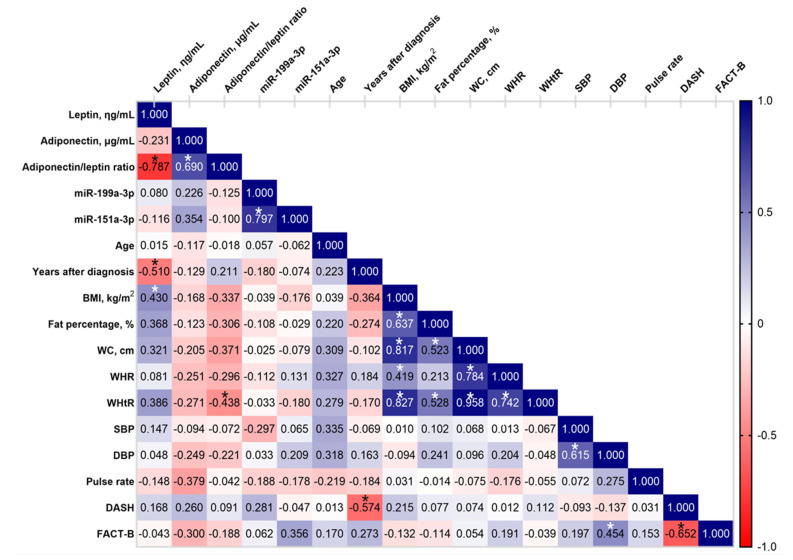
Correlation matrix of leptin, adiponectin level, and adiponectin/leptin ratio with miR-199a-3p, miR-151a-3p, and relevant variables of the study subjects in the BCRL group. As presented by the correlation range (right-hand bar), the darker blue color indicates a stronger positive correlation, the darker red color indicates a stronger negative correlation, and the white color indicates no correlation. The Pearson’s correlation coefficient (r) values for each correlation are shown in the rectangles and the asterisks represent a significant *p* value, *p* < 0.05, between the pairwise correlation. BMI body mass index, WC waist circumference; WHR waist-to-hip ratio; WHtR waist-to-height ratio; SBP systolic blood pressure; DBP diastolic blood pressure; DASH Disabilities of Arm, Shoulder, and Hand; FACT-B Functional Assessment of Cancer Therapy–Breast.

Based on the miRNA-sequencing and adipokine analysis, further correlation analysis was performed between circulating adipokines, miRNAs, and the relevant variables in the BCRL group of the combined cohort data. The circulating adipokines and adiponectin/leptin ratio were analyzed to identify their correlation with relevant characteristics in the BCRL group (Figure 8). Leptin concentration was negatively correlated with the adiponectin/leptin ratio (r_s_ = −0.787, *p* < 0.001) as well as the number of years after breast cancer diagnosis (r_s_ = 0.510, *p* = 0.018), and it was positively correlated with BMI (r_s_ = 0.430, *p* = 0.050). In contrast to leptin, circulating adiponectin only showed a strong positive correlation with the adiponectin/leptin ratio (r = 0.690, *p* < 0.001). Meanwhile, the adiponectin/leptin ratio demonstrated a moderate-negative correlation with WHtR (r_s_ = −0.438, *p* = 0.041). Additionally, no correlation was identified between either of the circulating adipokines with miR-199a-3p and miR-151a-3p (*p* < 0.05).

Meanwhile, a significant association was found between miR-199a-3p and miR-151a-3p (r_s_ = 0.797, *p* < 0.001), suggesting that both miRNAs have a similar profile of expression. There was no correlation between either of the miRNAs with variables including age, years after breast cancer diagnosis, BMI, fat percentage, WHR, waist circumference, WHtR, blood pressure, pulse rates, DASH, or FACT-B scores (Figure 8). Besides miRNAs, several variables were found to have an association with each other. A significant, moderate to strong positive association was observed between BMI and fat percentage (r_s_ = 0.637, *p* = 0.001), waist circumference (r_s_ = 0.419, *p* = 0.047), WHR (r_s_ = 0.827, *p* < 0.001), and WHtR (r_s_ = 0.817, *p* < 0.001). Fat percentage showed a positive correlation with waist circumference (r_s_ = 0.528, *p* = 0.010) and WHtR (r_s_ = 0.523, *p* = 0.010), and, as expected, WHR and WhtR demonstrated a strong correlation (r_s_ = 0.958, *p* < 0.001), as well as SBP and DBP (r_s_ = 0.615, *p* = 0.002). Additionally, DBP showed a moderate positive correlation with QoL or FACT-B scores (r_s_ = 0.454, *p* = 0.034). A strong negative correlation was also shown by years after diagnosis with DASH (r_s_ = –0.574, *p* = 0.004) and DASH with FACT-B scores (r_s_ = –0.652, *p* = 0.001).

## 3. Discussion

Arm lymphedema leads to limited movement of the upper quadrant extremity, discomfort including heaviness or tightness, pain, and disfiguration of body image, which all affect the physical and psychological well-being of the affected individual [3,21]. There is limited information on molecular signatures or reliable biomarkers for arm lymphedema which prompted the present study to identify circulating biomarkers for this chronic and incurable condition. Prior studies implicate several key pathological features that govern lymphedema including inflammation-induced lymphangiogenesis, immune−lymphatic dysfunction, tissue remodelling and fibrosis, and obesity-induced lymphedema [22,23]. Treatment for lymphedema involves management of the signs and symptoms in patients, such as manual lymphatic drainage or compression garments that aim to reduce arm swelling by ameliorating the lymphatic circulation [24,25]. As lymphedema is a multifactorial lymphatic disease and there is lack of molecular data on the key genes responsible for its onset, the development of molecular-based therapies for this condition remains a challenge. Therefore, most of the current experimental approaches are focused on the search for lymphedema biomarkers to facilitate risk assessment, diagnosis, prognosis, and treatment of the condition.

Characterization or profiling of biofluid is one of the first-line approaches in biomarker studies. In the present study, miRNA-sequencing of serum from BCRL compared to non-BCRL revealed a panel of deregulated miRNAs. Previous studies by Koc and colleagues identified four deregulated serum miRNAs (miR-1260a, miR-29b-3p, miR-130b-3p, and miR-660-5p), which distinguished BCRL patients (*n* = 7) from the combined healthy and non-BCRL group (*n* = 8) [26]. The expression of miR-29b and miR-144 were decreased in the serum of lymphedema patients, whereas miR-130b and miR-155 were elevated and had negative effects on adipocyte expansion [26]. These findings were in line with the present study where miR-144-5p was downregulated in the BCRL group, but no further validation was performed due to low read counts and a small fold change.

Guided by the initial results, miR-199a-3p and miR-151a-3p were further validated as the miRNAs that were the most abundant miRNAs in the sample and detectable by qPCR. Based on our previous work, it was found that breast cancer survivors who had diabetes mellitus have increased odds of developing early-onset lymphedema by 9.6-fold [15]. Therefore, associations between miR-151a-3p and miR-199a-3p with onset of lymphedema, diabetes mellitus, and hypertension were explored. It was found that both miRNAs were associated with the onset of lymphedema and miR-151a-3p was correlated with diabetes mellitus in the BCRL group.

Data published on circulating miR-151a-3p thus far are limited, but the association of miR-151a-3p with diabetes and obesity has been reported previously in the literature [26,27,28]. Akerman and colleagues identified that miR-151a-3p was negatively correlated with blood glucose, suggesting its lower concentration in children with type 1 diabetes [27]. A downregulation of circulating miR-151a-3p in the serum of obese patients has been demonstrated in several studies, indicating miR-151a-3p to be an obesity-specific miRNA [28,29]. Although obesity is a strong risk factor for BCRL in the present study, no correlation was observed between miR-151a-3p or miR-199a-3p and obesity parameters such as BMI, fat percentage, and WHR. Cumulatively, the expression of miR-151a-3p in this study might be attributable to the diabetic state of women with BCRL.

To date, the association of miR-199a-3p with lymphedema or lymphatic vasculature has never been reported. However, it has been demonstrated that reduced expression of miR-199a-3p in the peripheral blood of patients with type 2 diabetes mellitus was associated with endothelial vascular injury [30]. The overexpression of miR-199a-3p inhibited apoptosis in human umbilical vein endothelial cells (HUVECs), whereas transfection with miR-199a-3p mimics induced proliferation and migration of the cells by regulating the PI3K/AKT signalling pathway [30]. In support of this, Ahmed et al. reported the downregulation of miR-199a was correlated with downstream genes of PI3K/Akt signalling, VEGF, insulin-like growth factor 1 (IGF1), and fibroblast growth factor 1 (FGF1), which served as a regulator of the signalling cascade that regulates vascular endothelium protection [31]. Taken together, as the present study demonstrated a similar profile of expression for miR-151a-3p and miR-199a-3p, their downregulation in the early-onset lymphedema in BCRL group may regulate major diabetic-related pathways such as insulin resistance, PI3K/Akt signalling, MAPK signalling pathway, mTOR signalling, and phospholipase D signalling. More importantly, key genes of the respective pathways, such as AKT3, FGF2, FGF7, mitogen-activated protein kinase 4 (MAP3K4), MAP3K5, insulin receptor (INSR), AKT3, and mTOR, were also enriched in the analysis, thus explaining the link between diabetes and BCRL subjects in the present study.

The present study also demonstrated that the miRNAs Identified as being differentially regulated in BCRL regulate key genes such as TGIF (TGFB-induced homeobox factor), SMAD2, FGF2, FGF7, and fibronectin-1 (FN1), which are involved in the TGF-β signalling pathway, ECM-receptor interaction, and regulation of the actin cytoskeleton. One of the hallmarks of secondary lymphedema is fibrosis and tissue remodelling, which is characterized by the deposition of ECM proteins in the dermis and subcutaneous tissue, leading to hardening, inflexibility, and non-pitting edema with a *peau d’orange* look on the skin [22,32]. TGF-β signalling plays a pivotal role in accelerating fibrosis by regulating profibrotic factors such as collagen, laminin, fibronectin, and elastin in secondary lymphedema [17,33]. Additionally, an increased amount of collagen fibers in the edematous skin were found in lymphedema patients and animal models [22,34]. In relation to fibrosis, miR-199a-3p was reported to suppress cytokine signalling-7 (SOCS7) to upregulate signal transducer and activate transcription 3 (STAT3) activation, which is directly induced by TGF- β-driven p53 upregulation in renal fibrosis [35].

Higher BMI or obesity were found to be one of the strongest risk factors of BCRL, prompting the measurement of circulating adipokines as a biomarker of secondary lymphedema in this study. Circulating leptin levels were higher in women with BCRL, reflecting its association with higher BMI and susceptibility to fat tissue mass changes. Higher leptin levels are detected in the serum of BCRL cases and play a central role in altered adipose biology in vitro [17,26]. Leptin acts on receptors located in the central nervous system and has a direct action on various tissues including pancreatic B cells, suggesting its relationship with insulin [18,36]. Leptin antagonizes insulin, thereby causing the breakdown of insulin receptors, lowering insulin sensitivity, and elevating glucose synthesis, which subsequently leads to the development of obesity and diabetes mellitus [37,38,39]. Besides obesity, hyperleptinemia has been associated with hypertension and cardiovascular diseases [36,40]. Previous studies have identified that obese breast cancer patients are prone to develop postoperative lymphedema [5,41,42]. It was reported that lymphatic system dysfunction can lead to changes in lipid transport and fat deposition, thus leading to lymphedema and the development of a MetS [42,43]. Sato and colleagues reported that a high dose of leptin (100 ηg/mL) inhibited tube formation and cell proliferation of the human lymphatic endothelial cells in vitro [41]. Besides leptin, adipokines such as interleukin-6 have been reported to be increased in the serum in lymphedema patients to compensate for adipose tissue deposition as a response to chronic inflammation and lymphatic dysfunction [44].

Accumulating evidence of the association between obesity, hypertension, and diabetes mellitus in the present study provides a consensus on the involvement of MetS in BCRL. Several predictive markers for MetS have been proposed in previous literature, including the adiponectin/leptin ratio, WHR, and WHtR. The adiponectin/leptin ratio has been proposed to correlate better with insulin resistance and adipose tissue dysfunction than adiponectin or leptin alone [20]. Given that adiponectin and leptin work inversely, a decrease in the adiponectin/leptin ratio reflects alterations in obesity-associated metabolic disturbances. In this study, the adiponectin/leptin ratio was lower in the BCRL group (0.28) than in the non-BCRL group (0.41), but the risk of MetS in both groups was equal given that both groups showed values below <0.5, which indicates severe MetS risk. Another MetS indicator, WHtR, has been suggested to be a good indicator for MetS such as obesity and diabetes mellitus [45,46,47]. In the present study, the WHtR was identified to be higher in the BCRL group than in the non-BCRL group (0.67 vs. 0.57). One of the possible explanations for this higher ratio is due to the multiple conditions that lead to MetS, such as obesity, diabetes mellitus, and hypertension, that resembled by the participants in the BCRL group.

It is interesting to note that no correlation was observed between either of the adipokines with the circulating miRNAs analyzed in this study. It is hypothesized that those factors (adipokines and miRNAs) work independently in the development of lymphedema and its underlying conditions. One of the shortcomings of the present study was the fluctuation of the selected miRNAs in the discovery and validated cohort, which could be due to the condition of the individuals in the non-BCRL group that had similarities with BCRL cases such as hypertension, diabetes mellitus, or obesity. Despite that, the sample size applied in this study was unique and not readily available. Also, this study used one of the largest cohorts yet to study prospective transcriptomic biomarkers in BCRL. Although the cohort used for miRNA-sequencing were small, several miRNAs were significant and differentially expressed in the BCRL group, suggesting their potential to serve as biomarkers and these are worth validating in future lymphedema studies. Obviously, these results need to be verified in a larger group and further in vitro studies are warranted to determine the functions or biological effects of these miRNAs in secondary lymphedema.

## 4. Materials and Methods

### 4.1. Study Participants and Data Collection

Human ethics approval was obtained from The Ethics Committee for Research Involving Human Subjects, Universiti Putra Malaysia (JKEUPM-2020-013) and the Human Research Ethics Committee, University of Newcastle (H-2020-0125). The present study builds on our previous research and the classification of breast cancer survivors with BCRL and non-BCRL have been detailed in our previous work [15]. The inclusion criteria were as follows: female, aged above 18 years, Malaysian citizen, had undergone unilateral breast surgery, had completed all chemotherapy/radiotherapy, and had no current evidence of cancer during recruitment. A set of questionnaires was used to collect information on demographics, medical, and breast cancer history. Comorbidities, including hypertension and diabetes mellitus, were recorded as “yes” or “no”. The validated questionnaires, DASH and FACT-B [48], were also administered to assess the upper quadrant function and QoL of participants. Anthropometry measurements including body weight and fat percentage were recorded using a body composition monitor (Omron, Kyoto, Japan), whereas pulse rate and blood pressure were monitored using a blood pressure monitor (Microlife, Taipei, Taiwan). BMI (kg/m^2^), WHR (waist circumference, cm/hip circumference, cm), and WHtR (waist circumference, cm/height, cm) were calculated. A total of 6 mL blood was obtained from seven healthy volunteers, and 120/160 breast cancer survivors. However, seven samples from the breast cancer survivors were excluded due to hemolysis and the remaining 113 blood samples were processed to obtain the serum. Samples from breast cancer survivors were divided into BCRL (*n* = 23) and non-BCRL (*n* = 90). Blood tubes were placed at room temperature for 30 to 45 min to clot before centrifugation at 600× *g* for 15 min. Serum was collected, transferred into microcentrifuge tubes, and stored at −80 °C until further analysis. Samples were randomly classified into discovery and combined cohorts, which were then used for miRNA-sequencing, RT-qPCR, and ELISA.

### 4.2. RNA Extraction

For the small RNA-sequencing assay, a total of 600 μL of serum was used from each of the 21 samples (HC, *n* = 7, BCRL, *n* = 7 and non-BCRL, *n* = 7). Frozen serum was thawed on ice before the extraction of miRNA using the Nucleospin miRNA Plasma Kit (Macherey-Nagel, Duren, Germany). The amount of total RNA extracted was maintained at 50 ηg at 1 ηg/μL. Concentration, purity, and integrity of the total RNA were assessed using a small RNA chip on the Agilent 2100 Bioanalyzer (Agilent Technologies, Mulgrave VIC, Australia) at the Laboratory of Vaccines and Immuno-therapeutics, Institute of Bioscience, UPM. Meanwhile, for validation using RT-qPCR, total RNA was extracted from frozen serum (400 μL) using the miRNeasy Serum/Plasma Kit (QIAGEN, Germantown, MD, USA) according to the manufacturer’s instructions. The total RNA was eluted in 20 μL of RNase-free water. RNA concentration was measured by applying 2 μL of each sample onto the Epoch Microplate Spectrophotometer (Biotek Instrument, Winooski, VT, USA). Subsequently, all RNA samples were prepared to a final concentration of 15 ηg/μL for complementary DNA (cDNA) synthesis.

### 4.3. miRNA Sequencing (Library Preparation, Pre-Processing, and Data Analysis)

Library preparation and sequencing were outsourced to the Genome Institute of Singapore. MiRNA libraries were created with the QIASeq miRNA library kit (QIAGEN, Germany), which is suitable for low RNA yield samples. The libraries were then sequenced in paired-end fashion on a single flow cell lane on a NextSeq 550 (NextSeq Mid-Output kit 2 × 75 bp, Illumina, San Diego, CA, USA). The small RNA-sequencing runs yielded between 5,238,748 and 9,583,385 reads per sample in a single run and raw files were transferred with Amazon Web Services [49]. The demultiplexed raw read files were uploaded into the GeneGlobe Data Analysis Center (QIAGEN, Hilden, Germany) for quality control, alignment, and expression quantification. Briefly, processing of the raw reads included calibrating the miRbase entries, trimming off the 3′ adapter sequences, and removing low-quality reads. Following the trimming, the unique molecular indices (UMI) were identified and reads of less than ten base pairs (bp) were discarded. The remaining reads were then aligned to miRbase V21 [50] using Bowtie [51]. Following this, unmapped sequences were aligned to the human Genome Reference Consortium GRCh38 and associated UMIs were clustered together to identify possible novel miRNA molecules.

Differential expression analysis was performed in R Studio software (version 3.6.2) using the Bioconductor Package DESeq2 (version 1.30.15) [52] and read counts of cases were averaged across all samples in each group (HCs, non-BCRL, and BCRL). Differentially expressed miRNAs were identified with DESeq2 by comparison between HCs versus (vs.) non-BCRL and HCs vs. BCRL. The Benjamini and Hochberg procedure was applied to adjust for multiple testing. miRNAs with a false discovery rate (FDR) less than 5% (*p* < 0.05) and a log_2_(FC) > |1| were selected as significantly expressed miRNAs.

### 4.4. Validation of MicroRNA Using RT-qPCR

cDNA was generated using the Taqman Advanced miRNA cDNA synthesis kit (Applied Biosystems, Waltham, MA, USA) as per the manufacturer’s instructions. Briefly, the main steps of cDNA synthesis involved performing the poly(A) tailing reaction, adaptor ligation reaction, reverse transcription, and miR-Amp reaction. miRNA qPCR was performed using the following Taqman Advanced miRNA Assays (Applied Biosystems, Waltham, MA, USA), including hsa-miR-151a-3p (477919_mir) and hsa-miR-199a-3p (477961_mir). qPCR reactions were run in triplicate and the expression was calculated using the 2^−∆∆Ct^ method [53] and normalized to miR-16-5p (477860_mir), a potential endogenous control for circulating miRNAs in breast cancer [54].

### 4.5. MicroRNA Target Prediction, Gene Ontology, and Pathway Analysis

The selected miRNAs, miR-151a-3p and miR-199a-3p, were subjected to the mirDBV5 [55], miRDIP [56], and miRbase [50] databases to explore the target genes of the candidate miRNAs. To reduce false positive results, only the targets predicted with strong evidence from previous publications were retained in the results. For annotation and enrichment analysis, Enrichr [57,58] was used to analyze the associated KEGG pathways and GO of the predicted genes. The enrichment analysis of the miRNA target genes was based on the biological process (BP), molecular function (MF), and cellular component (CC). Significant genes were calculated based on default algorithms (Fisher’s exact and Z-scores) and pathways with adjusted *p* < 0.05 values were considered significantly enriched in the target candidate genes.

### 4.6. Measurement Using Adipokines Levels in ELISA

Serum adiponectin and leptin levels were analyzed using commercially available ELISA kits (Human Adiponectin (Abcam, Cambridge, UK) and Human Leptin (Thermo Fisher Scientific, Waltham, MA, USA)), according to the manufacturer’s instructions. Samples were diluted in 1:2000 for adiponectin and 1:100 for leptin analysis. All serum samples were tested in duplicates and run together with standards on each plate. The plates were placed in the Molecular Device Spectramax M5 microplate reader and the concentration was determined using standard curves built with Softmax Pro 7.1 software. The inter-assay and intra-assay variation were 10% and 15%, respectively.

### 4.7. Statistical Analysis

Statistical analysis was performed to compare the data between BCRL and the non-BCRL group (unless stated otherwise). Data were assessed for homogeneity and normally distributed data were presented as means with standard deviation (SD), whereas non-parametric tests were performed for non-normally distributed data and the results were interpreted as medians with interquartile range (IQR). Student’s *t* test, Mann–Whitney, and Chi-square tests assessed the demographic and clinical variable frequency between BCRL and non-BCRL in the combined cohorts. For miRNA-sequencing analysis, data from the BCRL and the non-BCRL groups were compared against the HCs to identify the differentially expressed miRNAs. Data obtained from RT-qPCR were compared between BCRL and non-BCRL groups in the discovery (*n* = 14) and validation cohorts (*n* = 99) by applying the Mann–Whitney test. The Mann–Whitney test was also applied to compare the data from the BCRL group in the combined cohort (*n* = 23) based on the onset of lymphedema and comorbidities (hypertension and diabetes mellitus). The onset of lymphedema was characterized based on the timing of the development of arm swelling, where <12 months after breast surgery was classified as early-onset lymphedema and ≥12 months after breast surgery was classified as late-onset lymphedema [59]. Adipokine levels and the adiponectin/leptin ratio were compared between the BCRL and non-BCRL groups using Mann–Whitney tests. Following that, Pearson’s correlation coefficient tests were performed between miRNAs, adipokines, and the adiponectin/leptin ratio, with relevant variables in the BCRL group including age, years after diagnosis, BMI (kg/m^2^), fat percentage, waist circumference (cm), WHR, WHtR, blood pressure (SBP and DBP), pulse rate, DASH, and FACT-B scores to observe whether associations exist with BCRL. A correlation coefficient (*r* value) of ≥|0.5| was considered strong, with moderate correlation defined as |0.5| > *r* ≥ |0.3|, and weak defined as *r* < |0.3| [60]. Meanwhile, an adiponectin/leptin ratio of >1.0 was regarded as normal, 0.5 to 1.0 as a moderately increased risk, and a ratio of <0.5 as a severely increased risk of MetS [20]. All statistical analyses were carried out using SPSS (IBM version 27, Armonk, NY, USA) and *p* < 0.05 was considered statistically significant. GraphPad Prism 9.0 (GraphPad Software Inc., San Diego, CA, USA) was used to generate graphs and diagrams of the findings.

## 5. Conclusions

In conclusion, findings from the present study have demonstrated that secondary lymphedema in breast cancer survivors showed an association with metabolic syndrome features including obesity, hypertension, and diabetes mellitus, as indicated by the circulating miRNA (miR-199a-3p, miR-151a-3p) and adipokine (leptin, adiponectin) analysis. The independent relationship between both miRNAs and leptin or adiponectin warrants new studies to further examine the contribution of other unexplored adipokines attributed to secondary lymphedema to improve the understanding of the pathophysiology that leads to the condition, thereby contributing to the development of a tool that can assess the risk of lymphedema in breast cancer survivors.

## Figures and Tables

**Table 1:** Characteristics of the participants for the combined cohort.

Characteristics	BCRL (*n* = 23)	Non-BCRL (*n* = 90)	OR [95% C.I.]	*p*-Value
Age, years ^a^	52.43 ± 7.70	51.61 ± 8.51		0.674
Years after diagnosis ^b^	4.00 (4.00)	4.00 (5.00)		0.528
BMI, kg/m^2 b^	29.30 (8.97)	26.71 (7.64)		0.073
Fat percentage, % ^b^	36.30 (9.50)	37.40 (7.30)		0.404
Waist circumference, cm ^b^	98.50 (18.00)	89.60 (15.95)		0.049 *
Waist-to-hip ratio ^a^	0.91 ± 0.07	0.88 ± 0.06		0.076
Waist-to-height ratio ^b^	0.62 (0.15)	0.57 (0.10)		0.049 *
Systolic blood pressure, mm/Hg ^b^, *n* = 110	132.50 (28.25)	125.50 (22.75)		0.167
Diastolic blood pressure, mm/Hg ^b^, *n* = 110	79.00 (18.75)	79.00 (13.75)		0.616
Heart rate, bpm ^b^	81.50 (20.75)	80.50 (15.50)		0.473
Arm disability scores (DASH) ^b^	40.00 (28.33)	17.37 (21.67)		<0.001 ^ϕ^
QoL scores (FACT-B) ^a^	107.76 ± 20.50	114.59 ± 17.00		0.103
**Co-morbidities**				
Hypertension ^c^	10 (43.47%)	15 (16.67%)	3.85 [1.42–10.39]	0.006 ^†^
Diabetes mellitus ^c^	7 (30.43 %)	10 (11.11%)	3.50 [1.16–10.56]	0.021 *

^a^*p* values of differences between means were calculated using independent Student’s *t* test and data are presented as means ± standard deviation (SD). ^b^ *p*-values of differences between groups were calculated using the Mann–Whitney U test, with data presented as medians (interquartile range, IQR). ^c^ Test of association using the Pearson’s chi-squared test, expressed as the number of subjects for each case and percentage. * *p* value < 0.05, ^†^ *p* value < 0.01, and ^ϕ^ *p* value < 0.001. Bpm beats per minute; C.I. confidence interval; DASH Disabilities of the Arm, Shoulder, and Hand; FACT-B Functional Assessment of Cancer Therapy–Breast; OR odd ratio; QoL quality of Life.

**Table 2:** Differentially expressed miRNAs in BCRL cases compared to HCs revealed by small RNA-sequencing. The FDR-adjusted *p*-value (Benjamini–Hochberg), *p* < 0.05 was considered statistically significant.

miRNA	Average Read Counts		*p*-Value	FDR-adj *p*-Value	Log_2_ FC	FC
	BCRL	HC				
hsa-miR-3613-5p	113.182	369.874	3.83 × 10^−8^	1.54 × 10^−5^	−1.708	0.306
hsa-miR-144-5p	57.294	173.178	6.96 × 10^−5^	4.66 × 10^−3^	−1.596	0.331
hsa-miR-206	9.864	29.701	3.32 × 10^−3^	3.81 × 10^−2^	−1.590	0.332
hsa-miR-676-5p	3.988	10.401	6.60 × 10^−4^	1.56 × 10^−2^	−1.383	0.383
hsa-miR-6804-5p	11.032	28.050	2.66 × 10^−4^	1.07 × 10^−2^	−1.346	0.393
hsa-miR-3122	8.868	21.873	5.50 × 10^−3^	4.91 × 10^−2^	−1.302	0.405
hsa-miR-3943	8.738	21.506	1.39 × 10^−4^	7.05 × 10^−3^	−1.299	0.406
hsa-miR-4785	4.925	10.897	4.45 × 10^−3^	4.59 × 10^−2^	−1.146	0.452
hsa-miR-7-5p	64.671	137.452	8.82 × 10^−4^	1.77 × 10^−2^	−1.088	0.471
hsa-miR-27a-5p	12.987	26.987	2.30 × 10^−3^	2.99 × 10^−2^	−1.055	0.481
hsa-miR-423-3p	602.444	294.668	4.36 × 10^−4^	1.46 × 10^−2^	1.032	2.044
hsa-miR-199b-3p	402.345	194.458	2.18 × 10^−3^	2.92 × 10^−2^	1.049	2.069
hsa-miR-584-5p	899.623	424.343	6.30 × 10^−4^	1.56 × 10^−2^	1.084	2.120
hsa-miR-1908-5p	60.460	28.355	4.76 × 10^−3^	4.73 × 10^−2^	1.092	2.132
hsa-miR-199a-3p	2601.828	1168.249	5.40 × 10^−3^	4.91 × 10^−2^	1.155	2.227
hsa-miR-139-3p	49.942	22.281	1.99 × 10^−3^	2.86 × 10^−2^	1.164	2.241
hsa-miR-625-5p	76.687	33.298	9.70 × 10^−4^	1.86 × 10^−2^	1.204	2.303
hsa-miR-28-5p	27.377	11.869	3.22 × 10^−3^	3.81 × 10^−2^	1.206	2.307
hsa-miR-1301-3p	51.522	21.774	6.71 × 10^−4^	1.56 × 10^−2^	1.243	2.366
hsa-miR-328-3p	124.036	52.264	1.12 × 10^−3^	2.05 × 10^−2^	1.247	2.373
hsa-miR-1307-3p	309.259	129.059	1.46 × 10^−4^	7.05 × 10^−3^	1.261	2.396
hsa-miR-151b/151a-5p	217.733	89.353	6.80 × 10^−4^	1.56 × 10^−2^	1.285	2.437
hsa-miR-326	50.869	20.848	1.98 × 10^−3^	2.86 × 10^−2^	1.287	2.440
hsa-miR-744-5p	248.133	101.532	4.87 × 10^−4^	1.50 × 10^−2^	1.289	2.444
hsa-miR-6126	121.629	46.948	1.59 × 10^−3^	2.62 × 10^−2^	1.373	2.591
hsa-miR-6872-3p	45.793	17.625	5.05 × 10^−3^	4.83 × 10^−2^	1.377	2.598
hsa-miR-151a-3p	2350.841	847.293	2.59 × 10^−7^	5.20 × 10^−5^	1.472	2.775
hsa-miR-370-3p	109.725	37.192	1.79 × 10^−3^	2.76 × 10^−2^	1.561	2.950
hsa-miR-382-5p	324.453	102.653	2.75 × 10^−3^	3.45 × 10^−2^	1.660	3.161
hsa-miR-199a-5p	77.776	22.796	7.49 × 10^−6^	1.00 × 10^−3^	1.771	3.412
hsa-miR-409-3p	251.053	72.922	1.25 × 10^−3^	2.18 × 10^−2^	1.784	3.443
hsa-miR-134-5p	119.347	33.867	6.99 × 10^−4^	1.56 × 10^−2^	1.817	3.524
hsa-miR-432-5p	521.532	132.257	7.92 × 10^−4^	1.68 × 10^−2^	1.979	3.943
hsa-miR-3135b	234.283	59.139	3.64 × 10^−5^	3.00 × 10^−3^	1.986	3.962
hsa-miR-4784	145.818	36.234	3.73 × 10^−5^	3.00 × 10^−3^	2.009	4.024
hsa-miR-431-5p	205.717	39.445	1.58 × 10^−4^	7.05 × 10^−3^	2.383	5.215

**Table 3:** Differentially expressed miRNAs in non-BCRL compared to HCs revealed by small RNA-sequencing. The FDR-adjusted *p* < 0.05 (Benjamini–Hochberg) was considered statistically significant.

miRNA	Average Read Counts		*p*-Value	FDR-adj *p*-Value	Log2 FC	FC
	Non-BCRL	HC				
hsa-miR-3147	8.299	24.182	3.46 × 10^−5^	9.63 × 10^−3^	−1.543	0.343
hsa-miR-92b-5p	16.151	41.999	5.89 × 10^−5^	9.63 × 10^−3^	−1.379	0.385
hsa-miR-6722-3p	14.501	33.790	3.42 × 10^−4^	2.79 × 10^−2^	−1.220	0.429
hsa-miR-3943	9.417	21.506	2.13 × 10^−4^	2.33 × 10^−2^	−1.191	0.438
hsa-miR-149-3p	11.406	26.042	6.01 × 10^−4^	3.93 × 10^−2^	−1.191	0.438
hsa-miR-6804-5p	12.564	28.050	1.11 × 10^−3^	4.52 × 10^−2^	−1.159	0.448
hsa-miR-604	10.165	21.617	9.02 × 10^−4^	4.35 × 10^−2^	−1.089	0.470
hsa-miR-941	76.161	27.029	9.31 × 10^−4^	4.35 × 10^−2^	1.495	2.818

**Table 4:** KEGG pathway analysis of miR-199a-3p and miR-151a-3p targets. The FDR-adjusted *p*-value (Benjamini–Hochberg), *p* < 0.05 was considered as statistically significant.

Pathway	No. of Overlapping Genes	Adjusted *p*-Value	Genes
PI3K-Akt signalling pathway	24	1.37 × 10^−5^	*YWHAE;ITGA3;INSR;ITGA1;FN1;PTEN;PPP2R2A;TSC1;LPAR4;FGF2;THBS1;MTOR;GHR;FGF7;PPP2R5E;ERBB4;AKT3;DDIT4;CHAD;ITGA8;ITGB8;PKN2;ITGA6;TP53*
MAPK signalling pathway	19	2.78 × 10^−4^	*MAP3K2;DUSP5;INSR;NLK;FGF2;RPS6KA3;RPS6KA6;FGF7;RPS6KA5;ERBB4;TAOK1;AKT3;GNA12;TAB2;TP53;LAMTOR3;CRK;MAP3K4;MAP3K5*
Regulation of actin cytoskeleton	16	2.78 × 10^−4^	*ITGA3;ITGA1;FN1;LPAR4;FGF2;MYLK4;GNA13;FGF7;CFL2;GNA12;ITGA8;ITGB8;ITGA6;CRK;PFN2;PAK4*
Central carbon metabolism in cancer	8	2.72 × 10^−3^	*PDHA1;AKT3;PTEN;TP53;HIF1A;HK2;MTOR;GLS*
Sphingolipid signalling pathway	10	3.70 × 10^−3^	*GNA13;PPP2R5E;PRKCE;AKT3;GNA12;PTEN;PPP2R2A;PLCB1;TP53;MAP3K5*
Focal adhesion	13	4.19 × 10^−3^	*ITGA3;ITGA1;FN1;PTEN;THBS1;MYLK4;AKT3;CHAD;ITGA8;ITGB8;ITGA6;CRK;PAK4*
ECM−receptor interaction	8	6.54 × 10^−3^	*ITGA3;ITGA1;CHAD;ITGA8;FN1;ITGB8;ITGA6;THBS1*
MicroRNAs in cancer	16	6.54 × 10^−3^	*PRKCE;DNMT3A;PTEN;HMGA2;THBS1;MTOR;GLS;FOXP1;RPS6KA5;MMP16;DDIT4;TIMP3;ZFPM2;TP53;CRK;PAK4*
Dilated cardiomyopathy	8	9.34 × 10^−3^	*ITGA3;ITGA1;ITGA8;ATP2A2;ITGB8;ITGA6;ADRB1;SLC8A1*
Phospholipase D signalling pathway	10	9.34 × 10^−3^	*GRM3;GNA13;INSR;AKT3;GNA12;TSC1;LPAR4;PLCB1;MTOR;RAPGEF4*
mTOR signalling pathway	10	1.00 × 10^−2^	*RPS6KA3;RPS6KA6;INSR;AKT3;DDIT4;PTEN;TSC1;LAMTOR3;LPIN2;MTOR*
Insulin resistance	8	1.38 × 10^−2^	*RPS6KA3;RPS6KA6;PRKCE;INSR;AKT3;PTEN;PTPRF;MTOR*
cGMP-PKG signalling	10	1.47 × 10^−2^	*GNA13;PRKCE;INSR;AKT3;GNA12;ATP2A2;ADRB1;PLCB1;SLC8A1;MYLK4*
Hyperthrophic cardiomyopathy	7	1.73 × 10^−2^	*ITGA3;ITGA1;ITGA8;ATP2A2;ITGB8;ITGA6;SLC8A1*
TGF-beta signalling pathway	7	1.84 × 10^−2^	*GREM1;SMAD2;TGIF2;ACVR1C;THBS1;ACVR2B;ACVR2A*

## Data Availability

The datasets generated during the current study are not available publicly due to domestic regulation of the institution. However, they are available upon request from the corresponding author.

## Supplementary Materials

The following supporting information is available online at: https://www.mdpi.com/article/10.3390/ijms231911359/s1.
