# Supplementary material for: Evaluation of Circulating MicroRNAs and Adipokines in Breast Cancer Survivors with Arm Lymphedema
Source: Int J Mol Sci. 2022 Sep 26;23(19):11359. doi: 10.3390/ijms231911359 (PMC9570352; doi:10.3390/ijms231911359)
Supplement: Supplementary file 1 [file ijms-23-11359-s001.zip › ijms-1881086-supplementary.pdf]

Table S1. Characteristics of the participants for discovery and validation cohort.

Discovery cohort				
**Characteristics**[a]	**HC (*n* = 7)**	**Non-BCRL (*n* = 7)**	**BCRL (*n* = 7)**	***P*-value**
Age, years	49.00 ± 7.25	48.71 ± 7.22	45.86 ± 7.98	0.692
BMI, kg/m^2	26.39 ± 5.04	30.19 ± 6.66	27.58 ± 6.15	0.494
Fat percentage, %	35.80 ± 5.81	37.55 ± 5.29	32.00 ± 6.63	0.229
Waist circumference, cm	92.65 ± 14.06	92.70 ± 10.19	87.37 ± 8.57	0.600
Waist-to-hip ratio	0.92 ± 0.05	0.90 ± 0.04	0.88 ± 0.04	0.445
Waist-to-height ratio	0.58 ± 0.09	0.59 ± 0.06	0.57 ± 0.07	0.758
Systolic blood pressure, mm/Hg	136.00 ± 18.56	128.14 ± 14.18	122.85 ± 23.93	0.456
Diastolic blood pressure, mm/Hg	80.14 ± 9.02	81.57 ± 15.59	79.85 ± 12.69	0.964
Heart rate, bpm	73.42 ± 9.01	78.28 ± 4.75	88.71 ± 16.67	0.059
Validation cohort				
**Characteristics**	**BCRL (*n* = 16)**	**Non-BCRL (*n* = 83)**	**OR [95% C.I.]**	***P*-value**
Age, years[b]	55.31 ± 5.70	51.86 ± 8.61		0.052
Years after diagnosis[c]	3.50 (4.00)	4.00 (5.00)		0.430
BMI, kg/m^2 [c]	30.03 (6.04)	26.67 (7.83)		0.013*
Fat percentage, %[c]	39.30 (9.70)	37.20 (6.90)		0.790
Waist circumference, cm[c]	102.15 (15.85)	89.40 (17.30)		0.004†
Waist-to-hip ratio[b]	0.92 ± 0.07	0.88 ± 0.07		0.029*
Waist-to-height ratio[c]	0.67 (0.09)	0.57 (0.10)		0.007†
Systolic blood pressure, mm/Hg[c], *n* = 96	133.00 (26.00)	126.00 (22.00)		0.051
Diastolic blood pressure, mm/Hg[c], *n* = 96	81.00 (18.00)	79.00 (13.50)		0.467
Heart rate, bpm[b]	83.13 ± 14.97	81.31 ± 12.10		0.607
Arm disability scores (DASH)[c]	40.83 (33.41)	17.50 (21.67)		<0.001ᶲ
QoL scores (FACT-B)[b]	107.87 ± 21.3	115.37 ± 17.0		0.125
**Comorbidities**				
Hypertension[d]	9 (56.25%)	13 (15.12%)	6.92 [2.19-21.89]	<0.001ᶲ
Diabetes mellitus[d]	7 (43.75%)	7 (8.14%)	8.44 [2.41-29.62]	<0.001ᶲ

[a]*P*-value of differences between means were calculated using one-way ANOVA which data are expressed as mean ± standard deviation (SD). [b]*P* value of differences between means were calculated using independent Student's t-test and data are presented as mean ± SD. [c]*P*-value of differences between groups were calculated from Mann–Whitney U test, with data presented as median (interquartile range, IQR). [d]Test of association using the Pearson's chi-squared test, expressed as number of subjects for each case and percentage. **p* value < 0.05, †*p* value < 0.01, and ᶲ*p* value < 0.001. Bpm beats per minute; C.I. confidence interval; DASH Disabilities of the Arm, Shoulder, and Hand; FACT-B Functional Assessment of Cancer Therapy – Breast; OR odd ratio; QoL – quality of Life

Table S2 Target gene prediction analysis

Gene	MiRNA	Integrated score
SERPINE2	hsa-miR-199a-3p	0.8774
MAP3K4	hsa-miR-199a-3p	0.8741
CELSR2	hsa-miR-199a-3p	0.8508
CDK17	hsa-miR-199a-3p	0.8397
QKI	hsa-miR-199a-3p	0.8338
ITGB8	hsa-miR-199a-3p	0.8233
ADAMTSL3	hsa-miR-199a-3p	0.8140
NAA25	hsa-miR-199a-3p	0.8132
VAMP3	hsa-miR-199a-3p	0.8119
KATNBL1	hsa-miR-199a-3p	0.8108
KLHL3	hsa-miR-199a-3p	0.8096
GNA12	hsa-miR-199a-3p	0.8052
ERBB4	hsa-miR-199a-3p	0.8047
NOVA1	hsa-miR-199a-3p	0.8021
CD2AP	hsa-miR-199a-3p	0.7972
CISD2	hsa-miR-199a-3p	0.7964
BCAR3	hsa-miR-199a-3p	0.7938
ADAMTS3	hsa-miR-199a-3p	0.7936
ZHX1	hsa-miR-199a-3p	0.7936
FXR1	hsa-miR-151a-3p	0.7921
ACVR2A	hsa-miR-199a-3p	0.7913
G3BP2	hsa-miR-199a-3p	0.7911
PFN2	hsa-miR-151a-3p	0.7906
FAM199X	hsa-miR-199a-3p	0.7905
CLASP2	hsa-miR-151a-3p	0.7857
DEPDC1B	hsa-miR-199a-3p	0.7850
PAWR	hsa-miR-199a-3p	0.7835
SCD	hsa-miR-199a-3p	0.7795
ZBTB18	hsa-miR-199a-3p	0.7775
NID2	hsa-miR-199a-3p	0.7769
CLK1	hsa-miR-151a-3p	0.7695
AK4	hsa-miR-199a-3p	0.7688
KTN1	hsa-miR-199a-3p	0.7676
ITPK1	hsa-miR-199a-3p	0.7667
PLEKHA3	hsa-miR-199a-3p	0.7661
ETNK1	hsa-miR-199a-3p	0.7660
PON2	hsa-miR-199a-3p	0.7645
NEDD4	hsa-miR-199a-3p	0.7630
VLDLR	hsa-miR-199a-3p	0.7614
KDM6A	hsa-miR-199a-3p	0.7601
CFL2	hsa-miR-199a-3p	0.7579
HIF1A	hsa-miR-151a-3p	0.7527
MEIS2	hsa-miR-199a-3p	0.7520
SLC20A2	hsa-miR-199a-3p	0.7504
ARL15	hsa-miR-199a-3p	0.7495
MTOR	hsa-miR-199a-3p	0.7495
SINHCAF	hsa-miR-199a-3p	0.7483
ZNF217	hsa-miR-199a-3p	0.7452
RB1	hsa-miR-199a-3p	0.7420
FXR1	hsa-miR-199a-3p	0.7413
AEBP2	hsa-miR-199a-3p	0.7398
TRA2B	hsa-miR-151a-3p	0.7396
ARHGEF3	hsa-miR-199a-3p	0.7393
MAP3K5	hsa-miR-199a-3p	0.7384
TGIF2	hsa-miR-199a-3p	0.7384
CELF2	hsa-miR-199a-3p	0.7379
RYBP	hsa-miR-151a-3p	0.7360
RBM47	hsa-miR-199a-3p	0.7357
SLC39A10	hsa-miR-199a-3p	0.7355
LCOR	hsa-miR-199a-3p	0.7336
PPP2R5E	hsa-miR-199a-3p	0.7331
PAK4	hsa-miR-199a-3p	0.7310
DDHD1	hsa-miR-199a-3p	0.7308
PRKCE	hsa-miR-199a-3p	0.7301
LRRC1	hsa-miR-199a-3p	0.7293
CPEB4	hsa-miR-199a-3p	0.7280
APLP2	hsa-miR-199a-3p	0.7273
TAB2	hsa-miR-199a-3p	0.7253
MCFD2	hsa-miR-199a-3p	0.7230

Gene	MiRNA	Integrated score
LRP2	hsa-miR-199a-3p	0.7229
LPAR4	hsa-miR-199a-3p	0.7221
GRK3	hsa-miR-199a-3p	0.7220
ZFPM2	hsa-miR-151a-3p	0.7217
YTHDF3	hsa-miR-151a-3p	0.7205
TACC2	hsa-miR-199a-3p	0.7179
C9orf40	hsa-miR-199a-3p	0.7175
TSPAN3	hsa-miR-199a-3p	0.7172
FUBP1	hsa-miR-199a-3p	0.7139
YWHAE	hsa-miR-199a-3p	0.7137
CCDC88A	hsa-miR-199a-3p	0.7137
FOXQ1	hsa-miR-199a-3p	0.7132
DNMT3A	hsa-miR-199a-3p	0.7110
DCBLD2	hsa-miR-199a-3p	0.7109
GREM1	hsa-miR-199a-3p	0.7108
CSRP2	hsa-miR-199a-3p	0.7073
KCTD16	hsa-miR-199a-3p	0.7064
RNGTT	hsa-miR-199a-3p	0.7064
KIAA0319L	hsa-miR-199a-3p	0.7060
RAP2A	hsa-miR-199a-3p	0.7053
CEP85L	hsa-miR-199a-3p	0.7046
FGF7	hsa-miR-199a-3p	0.7046
FBXW11	hsa-miR-199a-3p	0.7035
PNRC1	hsa-miR-199a-3p	0.7032
IL13RA1	hsa-miR-199a-3p	0.7027
ATAD1	hsa-miR-199a-3p	0.7023
DDIT4	hsa-miR-199a-3p	0.7017
RUNX1	hsa-miR-199a-3p	0.7013
PTPN3	hsa-miR-199a-3p	0.7004
CNEP1R1	hsa-miR-199a-3p	0.6994
CYB5R4	hsa-miR-199a-3p	0.6986
SLC6A5	hsa-miR-199a-3p	0.6985
PPP2R2A	hsa-miR-199a-3p	0.6971
TAOK1	hsa-miR-199a-3p	0.6962
IREB2	hsa-miR-199a-3p	0.6956
UPP2	hsa-miR-151a-3p	0.6954
PURB	hsa-miR-151a-3p	0.6944
SOCS5	hsa-miR-151a-3p	0.6941
PIGB	hsa-miR-199a-3p	0.6928
SDC2	hsa-miR-199a-3p	0.6926
WDR47	hsa-miR-199a-3p	0.6920
DLC1	hsa-miR-151a-3p	0.6901
MKRN1	hsa-miR-199a-3p	0.6898
FN1	hsa-miR-199a-3p	0.6894
IFFO2	hsa-miR-199a-3p	0.6885
UBE2W	hsa-miR-199a-3p	0.6884
PLEKHH1	hsa-miR-199a-3p	0.6876
ZHX2	hsa-miR-199a-3p	0.6864
RNF216	hsa-miR-199a-3p	0.6833
MBNL1	hsa-miR-199a-3p	0.6829
RABGAP1	hsa-miR-199a-3p	0.6822
CRK	hsa-miR-151a-3p	0.6819
MDGA2	hsa-miR-199a-3p	0.6811
DUSP5	hsa-miR-199a-3p	0.6804
ATRX	hsa-miR-199a-3p	0.6801
ITGA3	hsa-miR-199a-3p	0.6787
RORB	hsa-miR-199a-3p	0.6780
GOLGA4	hsa-miR-199a-3p	0.6779
NLK	hsa-miR-199a-3p	0.6777
NACC2	hsa-miR-199a-3p	0.6772
TMEM62	hsa-miR-199a-3p	0.6769
SLC49A4	hsa-miR-199a-3p	0.6768
RNF14	hsa-miR-199a-3p	0.6767
PLAG1	hsa-miR-199a-3p	0.6767
PHF6	hsa-miR-199a-3p	0.6753
PDE4B	hsa-miR-199a-3p	0.6753
LIN28B	hsa-miR-199a-3p	0.6726
EDEM3	hsa-miR-199a-3p	0.6721
TXLNG	hsa-miR-199a-3p	0.6720
AGO2	hsa-miR-151a-3p	0.6717
PLCB1	hsa-miR-199a-3p	0.6714

Gene	MiRNA	Integrated score
KLF12	hsa-miR-151a-3p	0.6714
GPBP1L1	hsa-miR-199a-3p	0.6710
COPS2	hsa-miR-199a-3p	0.6706
ZFAND5	hsa-miR-151a-3p	0.6697
TEAD1	hsa-miR-199a-3p	0.6696
SEMA3A	hsa-miR-199a-3p	0.6680
KDM3A	hsa-miR-199a-3p	0.6673
ADD3	hsa-miR-199a-3p	0.6673
ACOX1	hsa-miR-199a-3p	0.6662
CITED2	hsa-miR-199a-3p	0.6645
SOWAHC	hsa-miR-199a-3p	0.6631
SNN	hsa-miR-199a-3p	0.6631
ILRUN	hsa-miR-151a-3p	0.6630
TWIST1	hsa-miR-151a-3p	0.6623
ARID4B	hsa-miR-199a-3p	0.6616
CRIM1	hsa-miR-199a-3p	0.6611
UCK2	hsa-miR-199a-3p	0.6602
YAP1	hsa-miR-199a-3p	0.6599
ABCA1	hsa-miR-199a-3p	0.6597
ALX4	hsa-miR-199a-3p	0.6595
CREBRF	hsa-miR-199a-3p	0.6594
ADRB1	hsa-miR-199a-3p	0.6590
WAPL	hsa-miR-199a-3p	0.6589
STIM2	hsa-miR-199a-3p	0.6583
ACVR2B	hsa-miR-199a-3p	0.6582
ENOX2	hsa-miR-199a-3p	0.6581
NACC1	hsa-miR-199a-3p	0.6580
ESRP1	hsa-miR-199a-3p	0.6578
DIO2	hsa-miR-199a-3p	0.6574
PTPRZ1	hsa-miR-199a-3p	0.6563
RAPGEF4	hsa-miR-199a-3p	0.6561
UXS1	hsa-miR-199a-3p	0.6560
TANC1	hsa-miR-199a-3p	0.6531
AREL1	hsa-miR-199a-3p	0.6526
SECISBP2L	hsa-miR-199a-3p	0.6524
CHAD	hsa-miR-199a-3p	0.6519
AKT3	hsa-miR-151a-3p	0.6505
FBXO28	hsa-miR-199a-3p	0.6499
SIX1	hsa-miR-151a-3p	0.6494
SEC16B	hsa-miR-199a-3p	0.6493
NFIA	hsa-miR-199a-3p	0.6492
CNOT7	hsa-miR-199a-3p	0.6483
FAM104A	hsa-miR-151a-3p	0.6429
ATP2A2	hsa-miR-151a-3p	0.6358
PANK2	hsa-miR-151a-3p	0.6281
CASD1	hsa-miR-151a-3p	0.6276
NPAS3	hsa-miR-151a-3p	0.6194
MEX3C	hsa-miR-151a-3p	0.6179
EIF4G2	hsa-miR-151a-3p	0.6165
POU4F1	hsa-miR-151a-3p	0.6162
CHL1	hsa-miR-151a-3p	0.6131
PITPNA	hsa-miR-151a-3p	0.6118
PKN2	hsa-miR-151a-3p	0.6063
MAP1B	hsa-miR-151a-3p	0.5979
CALCR	hsa-miR-151a-3p	0.5783
CALD1	hsa-miR-151a-3p	0.5739
UBE2N	hsa-miR-151a-3p	0.5687
PAPOLG	hsa-miR-151a-3p	0.5673
TMX1	hsa-miR-151a-3p	0.5620
QKI	hsa-miR-151a-3p	0.5563
DEUP1	hsa-miR-151a-3p	0.5553
RANBP17	hsa-miR-151a-3p	0.5533
SMARCAD1	hsa-miR-151a-3p	0.5532
SEPTIN7	hsa-miR-151a-3p	0.5515
NAV3	hsa-miR-151a-3p	0.5496
NIPAL2	hsa-miR-151a-3p	0.5430
ZMAT1	hsa-miR-151a-3p	0.5421
RBM5	hsa-miR-151a-3p	0.5418
INSR	hsa-miR-151a-3p	0.5410
MAF	hsa-miR-151a-3p	0.5237
AGO3	hsa-miR-151a-3p	0.5237

Gene	MiRNA	Integrated score
RC3H1	hsa-miR-151a-3p	0.5156
CYLD	hsa-miR-151a-3p	0.5141
KBTBD2	hsa-miR-151a-3p	0.5140
CEP41	hsa-miR-151a-3p	0.5130
NAPEPLD	hsa-miR-151a-3p	0.5122
HK2	hsa-miR-151a-3p	0.5096
LAMTOR3	hsa-miR-151a-3p	0.5095
TANC2	hsa-miR-151a-3p	0.5090
NPL	hsa-miR-151a-3p	0.5087
GHR	hsa-miR-151a-3p	0.5071
LPIN2	hsa-miR-151a-3p	0.5063
TNIK	hsa-miR-151a-3p	0.5055
FBXL3	hsa-miR-151a-3p	0.5049
ZNF292	hsa-miR-151a-3p	0.5017
BRINP3	hsa-miR-151a-3p	0.5015
NUFIP2	hsa-miR-151a-3p	0.4991
CYP7B1	hsa-miR-151a-3p	0.4987
TMEM98	hsa-miR-151a-3p	0.4986
SLC4A7	hsa-miR-151a-3p	0.4981
CREBRF	hsa-miR-151a-3p	0.4948
TSC1	hsa-miR-151a-3p	0.4915
HMGA2	hsa-miR-151a-3p	0.4910
VAMP1	hsa-miR-151a-3p	0.4909
TNPO1	hsa-miR-151a-3p	0.4897
PCBP2	hsa-miR-151a-3p	0.4850
RPS6KA5	hsa-miR-151a-3p	0.4775
STRN3	hsa-miR-151a-3p	0.4753
SLC35F5	hsa-miR-151a-3p	0.4685
SEC63	hsa-miR-151a-3p	0.4679
CSNK1G1	hsa-miR-151a-3p	0.4673
PSME3	hsa-miR-151a-3p	0.4646
CHD9	hsa-miR-151a-3p	0.4645
RPRD2	hsa-miR-151a-3p	0.4627
MIER3	hsa-miR-151a-3p	0.4626
API5	hsa-miR-151a-3p	0.4570
PTGER3	hsa-miR-151a-3p	0.4540
BRWD1	hsa-miR-151a-3p	0.4535
PTPN12	hsa-miR-151a-3p	0.4496
FIGN	hsa-miR-151a-3p	0.4493
RPS6KA3	hsa-miR-151a-3p	0.4493
GLS	hsa-miR-151a-3p	0.4490
PLEKHA1	hsa-miR-151a-3p	0.4452
WDFY3	hsa-miR-151a-3p	0.4451
DBT	hsa-miR-151a-3p	0.4444
GRM3	hsa-miR-151a-3p	0.4423
TRIM33	hsa-miR-151a-3p	0.4416
SLC8A1	hsa-miR-151a-3p	0.4413
SP3	hsa-miR-151a-3p	0.4404
GOLM2	hsa-miR-151a-3p	0.4346
CALN1	hsa-miR-151a-3p	0.4344
MBTD1	hsa-miR-151a-3p	0.4337
MYLK4	hsa-miR-151a-3p	0.4331
PELI1	hsa-miR-151a-3p	0.4287
USP6NL	hsa-miR-151a-3p	0.4284
CUX1	hsa-miR-151a-3p	0.4278
KCNH8	hsa-miR-151a-3p	0.4275
ADNP	hsa-miR-151a-3p	0.4263
ANKRD44	hsa-miR-151a-3p	0.4263
NAA15	hsa-miR-151a-3p	0.4250
RAB22A	hsa-miR-151a-3p	0.4236
GABRB1	hsa-miR-151a-3p	0.4224
ASXL2	hsa-miR-151a-3p	0.4222
NIPBL	hsa-miR-151a-3p	0.4211
PTEN	hsa-miR-151a-3p	0.4187
ME1	hsa-miR-151a-3p	0.4184
NSD3	hsa-miR-151a-3p	0.4138
RFESD	hsa-miR-151a-3p	0.4134
ATAD2B	hsa-miR-151a-3p	0.4115
GAN	hsa-miR-151a-3p	0.4111
GLCCI1	hsa-miR-151a-3p	0.4100
KDM7A	hsa-miR-151a-3p	0.4090

Gene	MiRNA	Integrated score
SPAG9	hsa-miR-151a-3p	0.4059
PLCL2	hsa-miR-151a-3p	0.4059
PCDHAC1	hsa-miR-151a-3p	0.4052
MAN1A2	hsa-miR-151a-3p	0.4043
NEURL1B	hsa-miR-151a-3p	0.4037
RBM27	hsa-miR-151a-3p	0.4030
TAB3	hsa-miR-151a-3p	0.4025
HSPA4L	hsa-miR-151a-3p	0.4010
ALCAM	hsa-miR-151a-3p	0.3993
UHMK1	hsa-miR-151a-3p	0.3992
IDE	hsa-miR-151a-3p	0.3987
NFAT5	hsa-miR-151a-3p	0.3974
LRCH1	hsa-miR-151a-3p	0.3973
TP53	hsa-miR-151a-3p	0.3951
RNF20	hsa-miR-151a-3p	0.3933
GAREM1	hsa-miR-151a-3p	0.3929
FOXP1	hsa-miR-151a-3p	0.3921
OXR1	hsa-miR-151a-3p	0.3915
PGM3	hsa-miR-151a-3p	0.3913
MBNL2	hsa-miR-151a-3p	0.3907
GNA13	hsa-miR-151a-3p	0.3900
PLXNA4	hsa-miR-151a-3p	0.3873
SMAD2	hsa-miR-151a-3p	0.3870
SPATS2	hsa-miR-151a-3p	0.3865
WIPF2	hsa-miR-151a-3p	0.3850
LPGAT1	hsa-miR-151a-3p	0.3818
PPP1R16B	hsa-miR-151a-3p	0.3814
PTPRS	hsa-miR-151a-3p	0.3805
TIMP3	hsa-miR-151a-3p	0.3790
SAP30L	hsa-miR-151a-3p	0.3780
CKAP5	hsa-miR-151a-3p	0.3779
PDHA1	hsa-miR-151a-3p	0.3765
KCNC1	hsa-miR-151a-3p	0.3757
THBS1	hsa-miR-151a-3p	0.3754
SLC39A10	hsa-miR-151a-3p	0.3750
NUDT4	hsa-miR-151a-3p	0.3730
MTPN	hsa-miR-151a-3p	0.3726
RAB8B	hsa-miR-151a-3p	0.3708

Table S3 Top 10 enriched biological processes, molecular functions, and cellular components of miR-199a-3p and miR-151a-3p associated genes in BCRL group

	GO terms	Adjusted p-value
**Biological process**	regulation of gene expression (GO:0010468)	1.62E-08
	regulation of cellular macromolecule biosynthetic process (GO:2000112)	3.84E-05
	regulation of transcription, DNA-templated (GO:0006355)	6.07E-05
	regulation of nucleic acid-templated transcription (GO:1903506)	6.07E-05
	negative regulation of nucleic acid-templated transcription (GO:1903507)	1.95E-04
	negative regulation of cellular macromolecule biosynthetic process (GO:2000113)	2.57E-04
	positive regulation of transcription, DNA-templated (GO:0045893)	2.57E-04
	positive regulation of macromolecule metabolic process (GO:0010604)	2.71E-04
	negative regulation of transcription by RNA polymerase II (GO:0000122)	4.06E-04
	negative regulation of transcription, DNA-templated (GO:0045892)	6.92E-04
**Molecular function**	mRNA binding (GO:0003729)	5.74E-07
	protein serine/threonine kinase activity (GO:0004674)	5.45E-04
	histone deacetylase binding (GO:0042826)	0.002277
	transcription cis-regulatory region binding (GO:0000976)	0.019429
	mRNA 3'-UTR binding (GO:0003730)	0.020853
	disordered domain specific binding (GO:0097718)	0.020853
	sequence-specific double-stranded DNA binding (GO:1990837)	0.020853
	RNA binding (GO:0003723)	0.020853
	PDZ domain binding (GO:0030165)	0.020853
	protein phosphorylated amino acid binding (GO:0045309)	0.020853
**Cellular component**	nucleus (GO:0005634)	8.87E-08
	intracellular membrane-bounded organelle (GO:0043231)	1.63E-06
	axon (GO:0030424)	0.001367
	neuron projection (GO:0043005)	0.063685
	cytoplasmic stress granule (GO:0010494)	0.109715
	dendrite (GO:0030425)	0.109715
	activin receptor complex (GO:0048179)	0.109715
	protein kinase complex (GO:1902911)	0.109715
	RISC-loading complex (GO:0070578)	0.12868
	focal adhesion (GO:0005925)	0.158116

Table S4 Top 20 enriched KEGG pathway analysis of the associated genes in BCRL group

Term	Adjusted *p*-value	Genes
PI3K-Akt signaling pathway	1.37E-05	*YWHAE;ITGA3;INSR;ITGA1;FN1;PTEN;PPP2R2A;TSC1;LPAR4;FGF2;THBS1;MTOR;GHR;FGF7;PPP2R5E;ERBB4;AKT3;DDIT4;CHAD;ITGA8;ITGB8;PKN2;ITGA6;TP53*
MAPK signaling pathway	2.78E-04	*MAP3K2;DUSP5;INSR;NLK;FGF2;RPS6KA3;RPS6KA6;FGF7;RPS6KA5;ERBB4;TAOK1;AKT3;GNA12;TAB2;TP53;LAMTOR3;CRK;MAP3K4;MAP3K5*
Regulation of actin cytoskeleton	2.78E-04	*ITGA3;ITGA1;FN1;LPAR4;FGF2;MYLK4;GNA13;FGF7;CFL2;GNA12;ITGA8;ITGB8;ITGA6;CRK;PFN2;PAK4*
Central carbon metabolism in cancer	0.002742	*PDHA1;AKT3;PTEN;TP53;HIF1A;HK2;MTOR;GLS*
Sphingolipid signaling pathway	0.0037	*GNA13;PPP2R5E;PRKCE;AKT3;GNA12;PTEN;PPP2R2A;PLCB1;TP53;MAP3K5*
Focal adhesion	0.004191	*ITGA3;ITGA1;FN1;PTEN;THBS1;MYLK4;AKT3;CHAD;ITGA8;ITGB8;ITGA6;CRK;PAK4*
Shigellosis	0.006548	*FBXW11;PRKCE;SEPTIN7;HK2;MTOR;RPS6KA5;AKT3;UBE2N;TAB3;TAB2;PLCB1;TP53;CRK;PFN2*
ECM-receptor interaction	0.006548	*ITGA3;ITGA1;CHAD;ITGA8;FN1;ITGB8;ITGA6;THBS1*
MicroRNAs in cancer	0.006548	*PRKCE;DNMT3A;PTEN;HMGA2;THBS1;MTOR;GLS;FOXP1;RPS6KA5;MMP16;DDIT4;TIMP3;ZFPM2;TP53;CRK;PAK4*
Dilated cardiomyopathy	0.00934	*ITGA3;ITGA1;ITGA8;ATP2A2;ITGB8;ITGA6;ADRB1;SLC8A1*
Phospholipase D signaling pathway	0.00934	*GRM3;GNA13;INSR;AKT3;GNA12;TSC1;LPAR4;PLCB1;MTOR;RAPGEF4*
Proteoglycans in cancer	0.00934	*SMAD2;ERBB4;SDC2;AKT3;FN1;TWIST1;TIMP3;TP53;HIF1A;FGF2;THBS1;MTOR*
Human papillomavirus infection	0.00934	*RB1;ITGA3;ITGA1;FN1;PTEN;PPP2R2A;TSC1;THBS1;MTOR;PPP2R5E;AKT3;CHAD;ITGA8;ITGB8;ITGA6;TP53*
Arrhythmogenic right ventricular cardiomyopathy	0.009775	*ITGA3;ITGA1;ITGA8;ATP2A2;ITGB8;ITGA6;SLC8A1*
mTOR signaling pathway	0.010036	*RPS6KA3;RPS6KA6;INSR;AKT3;DDIT4;PTEN;TSC1;LAMTOR3;LPIN2;MTOR*
Insulin resistance	0.013881	*RPS6KA3;RPS6KA6;PRKCE;INSR;AKT3;PTEN;PTPRF;MTOR*
Pathways in cancer	0.014248	*RB1;SMAD2;ITGA3;PTGER3;FN1;PTEN;LPAR4;HIF1A;FGF2;MTOR;RUNX1;GNA13;FGF7;RPS6KA5;AKT3;GNA12;ITGA6;PLCB1;TP53;CRK;IL13RA1*
Apelin signaling pathway	0.014248	*SMAD2;GNA13;PRKCE;AKT3;TFAM;PLCB1;SLC8A1;MTOR;MYLK4*
cGMP-PKG signaling pathway	0.014763	*GNA13;PRKCE;INSR;AKT3;GNA12;ATP2A2;ADRB1;PLCB1;SLC8A1;MYLK4*
Hypertrophic cardiomyopathy	0.017311	*ITGA3;ITGA1;ITGA8;ATP2A2;ITGB8;ITGA6;SLC8A1*
